# Supplementary material for: Haplotype-level metabarcoding of freshwater macroinvertebrate species: A prospective tool for population genetic analysis
Source: PLoS One. 2023 Jul 24;18(7):e0289056. doi: 10.1371/journal.pone.0289056 (PMC10365294; doi:10.1371/journal.pone.0289056)
Supplement: S1 File — (DOCX) [file pone.0289056.s001.docx]

# Supplementary Information

**Haplotype-level metabarcoding of freshwater macroinvertebrate species: a prospective tool for population genetic analysis**

# *Authors and Affiliations*

Joeselle M. Serrana^1,2^ and Kozo Watanabe^1, *^

^1^ Center for Marine Environmental Studies (CMES), Ehime University, Matsuyama, Ehime, Japan

^2^ Faculty of Engineering, Graduate School of Science and Engineering, Ehime University, Matsuyama, Ehime, Japan

^*^ Correspondence: Kozo Watanabe; Molecular Ecology and Health Laboratory (MEcoH Lab), Center for Marine Environmental Studies (CMES), Ehime University, Bunkyo-cho 3, Matsuyama, Ehime 790-8577, Japan; [watanabe.kozo.mj@ehime-u.ac.jp](mailto:watanabe.kozo.mj@ehime-u.ac.jp)

# Supplementary Text

## *Sample information*

Twenty specimens from four species with known haplotypes from published population genetics studies (*Amphinemura decemseta*; Gamboa et al., 2019) of our current DNA barcoding projects (*Kamimuria tibialis*, *Eucapnopsis bulba*, and *Epeorus latifolium*) were chosen to create a mock sample in this study. The *Amphinemura decemseta* samples were collected from three sampling sites in Japan, i.e., Hokkaido, Honshu, and Shikoku (Gamboa et al., 2019). The other three species were collected from the Shigenobu River in Shikoku as part of monitoring activity along ten locations (site information listed in Serrana et al., 2019).

## *DNA extraction, amplification, and Sanger sequencing*

To generate the Sanger sequences, genomic DNA was extracted from the samples using the DNeasy Blood & Tissue Kit (Qiagen, Inc., Hilden, Germany) following manufacturer instructions. The 658-bp fragment of the cytochrome oxidase I (COI) region of the mitochondrial DNA was amplified using the universal Folmer primers, i.e., LCO1490 (GGTCAACAAATCATAAAGATATTGG) and HCO2198 (TAAACTTCAGGGTGACCAAAAAATCA) (Folmer et al., 1994). PCR amplification was performed with a T100TM Thermal Cycler (BioRad Ltd.). PCR cycling conditions were 30 s of initial denaturation at 98 °C, followed by 40 cycles of 10 s denaturation at 98°C, 30 s annealing at 38°C, 30 s extension at 72°C, and a final extension step of 5 min at 72°C. PCR products were purified by QIAquick PCR Purification Kit (Qiagen, Inc., Hilden, Germany), and DNA concentration was measured by Quantus™ Fluorometer using the QuantiFluor dsDNA System (Promega). Sanger sequencing was performed by the Eurofins Operon (Tokyo, Japan) in both directions. The forward and reverse reads were assembled and edited using CodonCode Aligner v 3.5 (Codon Code Corporation, Dedham, USA). The haplotypes were identified based on the Sanger sequence of the 658-bp Folmer region of the mitochondrial cytochrome c oxidase I (mtCOI) gene using DnaSP 6.0 (Rozas et al., 2017).

## References

Folmer, O., Black, M., Hoeh, W., Lutz, R., & Vrijenhoek, R. (1994). DNA primers for amplification of mitochondrial cytochrome c oxidase subunit I from diverse metazoan invertebrates. Molecular Marine Biology and Biotechnology, 3(5), 294-299.

Gamboa, M., Muranyi, D., Kanmori, S., & Watanabe, K. (2019). Molecular phylogeny and diversification timing of the Nemouridae family (Insecta, Plecoptera) in the Japanese Archipelago. PloS one, 14(1), e0210269.

Rozas, J., Ferrer-Mata, A., Sánchez-DelBarrio, J. C., Guirao-Rico, S., Librado, P., Ramos-Onsins, S. E., & Sánchez-Gracia, A. (2017). DnaSP 6: DNA sequence polymorphism analysis of large data sets. Molecular biology and evolution, 34(12), 3299-3302.

Serrana, J. M., Miyake, Y., Gamboa, M., & Watanabe, K. (2019). Comparison of DNA metabarcoding and morphological identification for stream macroinvertebrate biodiversity assessment and monitoring. Ecological Indicators, 101, 963-972.

# Supplementary Tables

**Supplementary Table 1.** Read abundance per read processing step.

| **Code** | **Cycle** | **Type** | **Raw** | **Merged** | **Matched** |
| --- | --- | --- | --- | --- | --- |
| 20B1 | 20 | Blank | 3 | 3 | - |
| 20B2 | 20 | Blank | 171 | 170 | - |
| 20B3 | 20 | Blank | 92 | 92 | - |
| 20X1 | 20 | Sample | 455 | 133 | 87 |
| 20X2 | 20 | Sample | 117 | 66 | 18 |
| 20X3 | 20 | Sample | 261 | 156 | 46 |
| 24B1 | 24 | Blank | 3 | 3 | - |
| 24B2 | 24 | Blank | 27 | 27 | - |
| 24B3 | 24 | Blank | - | - | - |
| 24X1 | 24 | Sample | 2,586 | 794 | 659 |
| 24X2 | 24 | Sample | 684 | 313 | 127 |
| 24X3 | 24 | Sample | 2,002 | 1,047 | 565 |
| 28B1 | 28 | Blank | 72 | 72 | - |
| 28B2 | 28 | Blank | 223 | 223 | 1 |
| 28B3 | 28 | Blank | 2 | - | - |
| 28X1 | 28 | Sample | 19,958 | 7,042 | 4,752 |
| 28X2 | 28 | Sample | 2,460 | 1,293 | 453 |
| 28X3 | 28 | Sample | 7,969 | 5,643 | 1,322 |
| 32B1 | 32 | Blank | 12 | 12 | - |
| 32B2 | 32 | Blank | 92 | 88 | 1 |
| 32B3 | 32 | Blank | 337 | 332 | - |
| 32X1 | 32 | Sample | 48,198 | 12,603 | 9,775 |
| 32X2 | 32 | Sample | 27,348 | 9,804 | 6,129 |
| 32X3 | 32 | Sample | 69,248 | 39,112 | 12,738 |
| 36B1 | 36 | Blank | 565 | 514 | 7 |
| 36B2 | 36 | Blank | 945 | 925 | 2 |
| 36B3 | 36 | Blank | 21,842 | 21,093 | 12 |
| 36X1 | 36 | Sample | 121,785 | 35,573 | 24,766 |
| 36X2 | 36 | Sample | 73,847 | 38,927 | 10,301 |
| 36X3 | 36 | Sample | 132,495 | 109,747 | 10,595 |
| 40B1 | 40 | Blank | 1,895 | 1,817 | 22 |
| 40B2 | 40 | Blank | 6,578 | 6,362 | 11 |
| 40B3 | 40 | Blank | 16,703 | 16,619 | - |
| 40X1 | 40 | Sample | 223,226 | 84,211 | 43,118 |
| 40X2 | 40 | Sample | 214,805 | 156,732 | 18,061 |
| 40X3 | 40 | Sample | 123,912 | 110,544 | 6,964 |
| 44B1 | 44 | Blank | 3,009 | 2,973 | - |
| 44B2 | 44 | Blank | 7,223 | 7,183 | 2 |
| 44B3 | 44 | Blank | 20,080 | 20,050 | - |
| 44X1 | 44 | Sample | 153,938 | 100,933 | 18,184 |
| 44X2 | 44 | Sample | 178,176 | 156,334 | 7,490 |
| 44X3 | 44 | Sample | 240,113 | 217,600 | 11,321 |
| 48B1 | 48 | Blank | 6,316 | 6,278 | - |
| 48B2 | 48 | Blank | 12,134 | 12,097 | - |
| 48B3 | 48 | Blank | 33,364 | 33,274 | - |
| 48X1 | 48 | Sample | 205,721 | 145,475 | 21,946 |
| 48X2 | 48 | Sample | 293,895 | 273,750 | 6,271 |
| 48X3 | 48 | Sample | 1,436,640 | 1,391,143 | 20,677 |
| 52B1 | 52 | Blank | 14,097 | 13,897 | - |
| 52B2 | 52 | Blank | 51,069 | 49,853 | 8 |
| 52B3 | 52 | Blank | 69,592 | 69,044 | - |
| 52X1 | 52 | Sample | 428,750 | 310,748 | 37,187 |
| 52X2 | 52 | Sample | 763,312 | 664,086 | 30,686 |
| 52X3 | 52 | Sample | 519,213 | 506,659 | 3,546 |
| 56B1 | 56 | Blank | 15,770 | 15,594 | 1 |
| 56B2 | 56 | Blank | 36,971 | 36,560 | 3 |
| 56B3 | 56 | Blank | 68,715 | 68,349 | - |
| 56X1 | 56 | Sample | 356,334 | 286,055 | 21,953 |
| 56X2 | 56 | Sample | 502,448 | 465,091 | 9,957 |
| 56X3 | 56 | Sample | 1,075,444 | 1,059,422 | 3,078 |
| 60B1 | 60 | Blank | 24,423 | 24,189 | 2 |
| 60B2 | 60 | Blank | 51,933 | 51,411 | 1 |
| 60B3 | 60 | Blank | 47,864 | 47,595 | 8 |
| 60X1 | 60 | Sample | 395,742 | 330,643 | 21,676 |
| 60X2 | 60 | Sample | 1,067,895 | 950,638 | 38,811 |
| 60X3 | 60 | Sample | 2,518,214 | 2,460,087 | 12,236 |
| 64B1 | 64 | Blank | 22,301 | 22,169 | - |
| 64B2 | 64 | Blank | 31,967 | 31,853 | 1 |
| 64B3 | 64 | Blank | 58,676 | 58,505 | 1 |
| 64X1 | 64 | Sample | 349,248 | 307,822 | 15,315 |
| 64X2 | 64 | Sample | 1,237,853 | 1,166,596 | 28,898 |
| 64X3 | 64 | Sample | 1,282,505 | 1,270,168 | 2,874 |
| **Total** | | | **14,701,863** | **13,296,216** | **462,665** |

**Supplementary Table 2.** Absolute read abundance of the zero-radius operational taxonomic units (ZOTUs) of each PCR cycle assessed in the study.

| **Sample** | | **ZOTU01** | **ZOTU02** | **ZOTU03** | **ZOTU04** | **ZOTU05** | **ZOTU06** | **ZOTU07** | **ZOTU08** | **ZOTU09** | **ZOTU10** | **ZOTU11** | **ZOTU12** | **ZOTU13** | **ZOTU14** |
| --- | --- | --- | --- | --- | --- | --- | --- | --- | --- | --- | --- | --- | --- | --- | --- |
| PCR Cycles | 20XR1 | 61 | 5 | 4 | - | 7 | 3 | 3 | 1 | - | - | 3 | - | - | - |
|  | 20XR2 | 16 | 1 | 1 | - | - | - | - | - | - | - | - | - | - | - |
|  | 20XR3 | 45 | - | - | - | - | 1 | - | - | - | - | - | - | - | - |
|  | 24XR1 | 465 | 15 | 22 | 12 | 46 | 32 | 20 | 5 | 1 | 2 | 34 | 1 | 1 | 3 |
|  | 24XR2 | 115 | 2 | 1 | 1 | 3 | - | 2 | 2 | - | - | 1 | - | - | - |
|  | 24XR3 | 533 | 5 | 2 | 1 | 6 | 2 | 1 | 1 | 1 | - | 13 | - | - | - |
|  | 28XR1 | 2,724 | 276 | 229 | 152 | 368 | 348 | 186 | 62 | 18 | 32 | 294 | 24 | 26 | 13 |
|  | 28XR2 | 424 | 4 | 4 | - | 5 | 1 | 1 | 5 | - | - | 9 | - | - | - |
|  | 28XR3 | 1,248 | 9 | 7 | 2 | 5 | 3 | - | 5 | 1 | - | 42 | - | - | - |
|  | 32XR1 | 3,696 | 805 | 785 | 525 | 1,031 | 947 | 608 | 208 | 71 | - | 860 | 68 | 116 | 55 |
|  | 32XR2 | 3,764 | 392 | 242 | 135 | 606 | 319 | 210 | 79 | 17 | 20 | 286 | 16 | 27 | 16 |
|  | 32XR3 | 10,064 | 398 | 242 | 139 | 541 | 315 | 59 | 22 | 11 | 13 | 888 | 12 | 21 | 13 |
|  | 36XR1 | 6,184 | 2,343 | 2,255 | 1,986 | 2,252 | 2,485 | 1,795 | 1,222 | 488 | 642 | 1,550 | 532 | 658 | 374 |
|  | 36XR2 | 8,716 | 61 | 16 | 1 | 14 | 5 | - | 15 | - | - | 1,472 | - | - | 1 |
|  | 36XR3 | 9,420 | 391 | 206 | 4 | 20 | 13 | 21 | 165 | - | - | 349 | 1 | 5 | - |
|  | 40XR1 | 7,490 | 4,312 | 4,080 | 4,138 | 3,662 | 4,267 | 3,001 | 3,376 | 1,303 | 1,370 | 2,514 | 1,108 | 1,429 | 1,068 |
|  | 40XR2 | 11,781 | 1,377 | 562 | 57 | 735 | 229 | 424 | 350 | 6 | 6 | 2,396 | 42 | 96 | - |
|  | 40XR3 | 6,925 | - | - | - | - | 2 | - | 29 | - | - | 8 | - | - | - |
|  | 44XR1 | 3,231 | 2,544 | 1,842 | 1,759 | 2,287 | 2,366 | 1,465 | 573 | 217 | 448 | 612 | 228 | 393 | 219 |
|  | 44XR2 | 6,120 | 416 | 93 | 9 | 66 | 9 | 4 | 22 | - | 1 | 749 | - | 1 | - |
|  | 44XR3 | 10,612 | 40 | 64 | 1 | 34 | 6 | 4 | 111 | 1 | - | 448 | - | - | - |
|  | 48XR1 | 4,241 | 2,717 | 1,961 | 2,258 | 2,968 | 2,442 | 1,558 | 1,244 | 256 | 490 | 980 | 231 | 371 | 229 |
|  | 48XR2 | 5,052 | 21 | 23 | - | 1 | - | 5 | 16 | 1 | - | 1,151 | - | 1 | - |
|  | 48XR3 | 9,720 | 2,935 | 1,465 | 1,149 | 2,033 | 212 | 66 | 1,455 | 73 | 24 | 1,490 | 24 | 14 | 17 |
|  | 52XR1 | 5,899 | 5,046 | 3,763 | 3,113 | 4,243 | 4,283 | 2,922 | 2,222 | 462 | 1,761 | 1,360 | 598 | 926 | 589 |
|  | 52XR2 | 10,055 | 3,284 | 2,571 | 1,150 | 4,287 | 3,351 | 2,374 | 532 | 112 | 297 | 2,132 | 130 | 301 | 110 |
|  | 52XR3 | 3,325 | - | 2 | - | 1 | 3 | - | 31 | 1 | - | 182 | - | 1 | - |
|  | 56XR1 | 4,114 | 2,702 | 2,166 | 2,000 | 2,605 | 2,551 | 1,794 | 1,384 | 289 | 440 | 1,005 | 225 | 392 | 286 |
|  | 56XR2 | 7,480 | 241 | 113 | - | 242 | 7 | 7 | 47 | 2 | 1 | 1,815 | - | 1 | 1 |
|  | 56XR3 | 2,652 | 183 | 69 | 2 | 8 | 2 | 2 | 105 | 1 | - | 54 | - | - | - |
|  | 60XR1 | 4,363 | 2,823 | 2,666 | 1,850 | 2,867 | 2,424 | 1,566 | 888 | 185 | 449 | 946 | 156 | 324 | 169 |
|  | 60XR2 | 8,085 | 4,792 | 4,354 | 3,508 | 2,623 | 4,413 | 2,799 | 1,669 | 488 | 1,130 | 3,196 | 497 | 795 | 462 |
|  | 60XR3 | 4,990 | 700 | 1,720 | 860 | 2,383 | 267 | 188 | 553 | 76 | 40 | 418 | 7 | 19 | 15 |
|  | 64XR1 | 3,297 | 1,927 | 1,557 | 1,229 | 1,983 | 1,935 | 1,262 | 563 | 140 | 380 | 615 | 138 | 202 | 87 |
|  | 64XR2 | 5,110 | 3,796 | 2,983 | 2,517 | 3,558 | 5,041 | 2,680 | 998 | 277 | 418 | 707 | 190 | 444 | 179 |
|  | 64XR3 | 2,357 | 270 | 85 | 4 | 15 | 8 | 13 | 37 | 2 | 1 | 76 | - | 6 | - |
| Negative Control | 20BR1 | - | - | - | - | - | - | - | - | - | - | - | - | - | - |
|  | 20BR2 | - | - | - | - | - | - | - | - | - | - | - | - | - | - |
|  | 20BR3 | - | - | - | - | - | - | - | - | - | - | - | - | - | - |
|  | 24BR1 | - | - | - | - | - | - | - | - | - | - | - | - | - | - |
|  | 24BR2 | - | - | - | - | - | - | - | - | - | - | - | - | - | - |
|  | 24BR3 | - | - | - | - | - | - | - | - | - | - | - | - | - | - |
|  | 28BR1 | - | - | - | - | - | - | - | - | - | - | - | - | - | - |
|  | 28BR2 | - | - | - | 1 | - | - | - | - | - | - | - | - | - | - |
|  | 28BR3 | - | - | - | - | - | - | - | - | - | - | - | - | - | - |
|  | 32BR1 | - | - | - | - | - | - | - | - | - | - | - | - | - | - |
|  | 32BR2 | - | - | - | 1 | - | - | - | - | - | - | - | - | - | - |
|  | 32BR3 | - | - | - | - | - | - | - | - | - | - | - | - | - | - |
|  | 36BR1 | 2 | - | - | 5 | - | - | - | - | - | - | - | - | - | - |
|  | 36BR2 | - | - | - | 1 | - | - | - | - | 1 | - | - | - | - | - |
|  | 36BR3 | - | - | - | 1 | - | 1 | - | - | 9 | - | 1 | - | - | - |
|  | 40BR1 | 8 | - | - | 13 | - | - | - | - | 1 | - | - | - | - | - |
|  | 40BR2 | - | - | - | 4 | - | - | - | - | 7 | - | - | - | - | - |
|  | 40BR3 | - | - | - | - | - | - | - | - | - | - | - | - | - | - |
|  | 44BR1 | - | - | - | - | - | - | - | - | - | - | - | - | - | - |
|  | 44BR2 | - | - | - | 2 | - | - | - | - | - | - | - | - | - | - |
|  | 44BR3 | - | - | - | - | - | - | - | - | - | - | - | - | - | - |
|  | 48BR1 | - | - | - | - | - | - | - | - | - | - | - | - | - | - |
|  | 48BR2 | - | - | - | - | - | - | - | - | - | - | - | - | - | - |
|  | 48BR3 | - | - | - | - | - | - | - | - | - | - | - | - | - | - |
|  | 52BR1 | - | - | - | - | - | - | - | - | - | - | - | - | - | - |
|  | 52BR2 | - | - | - | 6 | - | - | - | - | - | - | 2 | - | - | - |
|  | 52BR3 | - | - | - | - | - | - | - | - | - | - | - | - | - | - |
|  | 56BR1 | - | - | - | 1 | - | - | - | - | - | - | - | - | - | - |
|  | 56BR2 | - | - | - | 3 | - | - | - | - | - | - | - | - | - | - |
|  | 56BR3 | - | - | - | - | - | - | - | - | - | - | - | - | - | - |
|  | 60BR1 | - | - | - | 2 | - | - | - | - | - | - | - | - | - | - |
|  | 60BR2 | - | - | - | 1 | - | - | - | - | - | - | - | - | - | - |
|  | 60BR3 | - | - | - | - | - | - | - | 8 | - | - | - | - | - | - |
|  | 64BR1 | - | - | - | - | - | - | - | - | - | - | - | - | - | - |
|  | 64BR2 | - | - | - | 1 | - | - | - | - | - | - | - | - | - | - |
|  | 64BR3 | - | - | - | - | - | - | - | - | - | 1 | - | - | - | - |
| **Total** | | **174,374** | **44,833** | **36,155** | **28,562** | **41,505** | **38,292** | **25,040** | **17,997** | **4,500** | **7,965** | **28,655** | **4,228** | **6,570** | **3,906** |

**Supplementary Table 3.** Taxonomic match of the zero-radius operational taxonomic units (ZOTUs) from the DNA metabarcoding sequences against the 658-bp mtCOI Sanger sequences.

| ZOTU | Order | Family | Genus | Species | Sanger Haplotype |
| --- | --- | --- | --- | --- | --- |
| ZOTU01 | Plecoptera | Perlidae | *Kamimuria* | *Kamimuria tibialis* | KT7 |
| ZOTU02 | Plecoptera | Capniidae | *Eocapnosis* | *Eucapnopsis bulba* | EB5 |
| ZOTU03 | Plecoptera | Capniidae | *Eocapnosis* | *Eucapnopsis bulba* | EB4 |
| ZOTU04 | Plecoptera | Perlidae | *Kamimuria* | *Kamimuria tibialis* | KT2 |
| ZOTU05 | Plecoptera | Perlidae | *Kamimuria* | *Kamimuria tibialis* | KT5 |
| ZOTU06 | Ephemeroptera | Heptageniidae | *Epeorus* | *Epeorus latifolium* | EL5 |
| ZOTU07 | Ephemeroptera | Heptageniidae | *Epeorus* | *Epeorus latifolium* | EL4 |
| ZOTU08 | Plecoptera | Capniidae | *Eocapnosis* | *Eucapnopsis bulba* | EB3 |
| ZOTU09 | Plecoptera | Perlidae | *Kamimuria* | *Kamimuria tibialis* | KT4 |
| ZOTU10 | No Match | No Match | No Match | No Match | No Match |
| ZOTU11 | Plecoptera | Perlidae | *Kamimuria* | *Kamimuria tibialis* | KT6 |
| ZOTU12 | Ephemeroptera | Heptageniidae | *Epeorus* | *Epeorus latifolium* | EL2 |
| ZOTU13 | Ephemeroptera | Heptageniidae | *Epeorus* | *Epeorus latifolium* | EL3 |
| ZOTU14 | No Match | No Match | No Match | No Match | No Match |
