# Supplementary material for: Haplotype-level metabarcoding of freshwater macroinvertebrate species: A prospective tool for population genetic analysis
Source: PLoS One. 2023 Jul 24;18(7):e0289056. doi: 10.1371/journal.pone.0289056 (PMC10365294; doi:10.1371/journal.pone.0289056)
Supplement: Attachment — Submitted filename: Response to Reviewers.docx [file pone.0289056.s002.docx]

# Editor’s Comments:

1. *Overall, I would like to encourage the authors to pay attention to comments related to technical aspects, which, if addressed, will help improving the manuscript. This includes clarification and/or rephrasing of statements regarding preparation of mock communities, high ‘loss’ of sequences in the pipeline, justification for thermal conditions and for very small mock community with low taxonomic diversity, as well as the discussion of some of the results (further discussion why some of the haplotypes in the mock community were not detected, and how to overcome this issue; future directions).*

**Response:**  We thank the editor for the additional comments and feedback on our manuscript. This version has been revised accordingly. In particular, we have rephrased or added statements regarding the preparation of mock communities (Reviewer #1 – Response #3), high loss of sequences (Lines 145-147), justification of thermal conditions (Lines 225-235), low taxonomic diversity (Reviewer #3 – Response #2), and additional discussion of some results (Lines 204-214), and future direction (revised Conclusion section) in this revised version.

2. *As such, it would be helpful to include a closing paragraph summarizing the perspectives and recommendations to further develop the approach.*

**Response:** A paragraph was added in the Discussion section for *DNA metabarcoding for haplotype-level inference* of the manuscript accordingly. Please refer to Reviewer #2 – Comment #1 and Reviewer #3 – Comment #8.

3. *Finally, I recommend the authors pay special attention to concerns raised by reviewer 2 regarding the motivation of this study. While novelty and/or perceived impact of a study is not a criterium for publication in Plos ONE, haplotype-level metabarcoding approaches have recently been explored in the referenced study by Elbrecht et al. (2018). In this light, I agree with the reviewer and recommend a slight reframing of the overall study focus (e.g., development of a more time and cost-effective method using NGS to barcode large numbers of bulk samples). Framed in this context, the study would benefit by reaching a broader audience and increasing its visibility.*

**Response:** We thank the Editor and the reviewers for this valuable comment. We really appreciate your concerns about reframing the narrative of our study to cater to a broader audience. Please refer to the response to Reviewer #2 on the necessary additions and edits to this version of the manuscript.

# Reviewer #1:

(Changes highlighted in **BLUE** font on the revised version of the manuscript)

1. *The presented paper aims to evaluate whether haplotype information can be obtained from DNA metabarcoding studies for population genetic analyses. For that, the authors used a mock community of known haplotypes and tested the influence of input DNA concentration, and the number PCR cycles on the detection ability of the known haplotypes. Interestingly, they found that the relative abundance OTUs were stable with increasing number of PCR cycles. The detection of a few haplotypes failed, which is probably due to their low input DNA concentration. The manuscript is well written, and the data and analyses results support the drawn conclusions. All data is available, and links with analysis scripts are functional. Some points need to be addressed and clarified before the paper can be accepted, but in general the study is scientifically sound and therefore, I recommend it for publication after minor revision.*

**Response:**  We appreciate the reviewer's remarks on the potential of our study. This version of the manuscript has been revised accordingly thanks to the reviewer’s generous comments and suggestions.

2. *Introduction: Line 50: Please correct it to‚ is typically not explored’.*

**Response:**  Revised to “…is typically not explored” accordingly (Line 39).

3. *Methods: Line 104: Please rephrase this sentence. You did not prepare different concentrations of each haplotype; each haplotype was present exactly one time in a specific concentration, and the 20 haplotypes were present in varying concentrations. Reading this sentence, I had the expectation that several mock communities were prepared, which is not the case.*

**Response:**  Rephrased to “To examine if DNA template concentration influences the detection of true haplotypes from the mock sample, haplotypes for each species were prepared with different concentrations before pooling their DNA samples in one tube for amplicon library preparation.” (Lines 78-81).

4. *Line 112-114: These fusion primers have an inline shift, which allow sample tagging. I assume these were used and samples were tagged at this step, otherwise you could not demultiplex 72 after pooling and sequencing. Please add this information for readers convenience.*

**Response:**  Added the statement, “Each primer has unique inline shifts and Illumina adapters attached to the 5’-end for sample tagging and multiplexing” (Lines 94-95).

5. *Line 117: These are not the PCR cycling conditions, which are typically used for BF2/BR2. Why did the authors choose this temperature regime? The higher annealing temperature makes the reaction more specific i.e., mismatches between primer and DNA template are less tolerated. This step of the PCR is a competition of molecules. Can the increased annealing temperature affect the detection rate of low concentration haplotypes?*

**Response:** We agree with the reviewer that the typical PCR annealing temperature for the BF2/BR2 primers, i.e., 50°C, performs well on a wide variety of macroinvertebrates, both in theory and practice (e.g., Zizka et al., 2019; Leese et al., 2021). We also agree that a higher annealing temperature makes the reaction more specific. Hence, for the one-step PCR, we performed in this study, we optimized the PCR conditions first with a gradient PCR and chose 55°C as the best annealing temperature for each individual in the mock sample. Still, we agree with the reviewer that the increased annealing temperature might affect the detection of low-concentration haplotypes when pooled as one.

We added a limitation statement and a short discussion on annealing temperature influence in our results, “Additionally, we used a PCR annealing temperature of 55°C chosen after a temperature gradient PCR of each individual, which was also the optimal condition used from a previous bulk metabarcoding study on freshwater macroinvertebrate communities (Serrana et al., 2022). This temperature is relatively higher than the typical conditions, i.e., 50°C, previously used for the BF2 and BR2 fusion primers (e.g., Zizka et al., 2019; Leese et al., 2021). This could have also led to the non-detection of the low-concentration haplotypes (e.g., AD1, EB1, EL1 with 0.01 ng/uL) in the mock sample. Although previous studies have reported that annealing temperatures from 40–56°C did not have a universal effect on taxonomic recovery (Elbrecht et al., 2019), lowering the annealing temperature could have recovered the haplotypes with low DNA template concentration (Aylagas et al., 2016). Hence, we recommend further assessment of the PCR conditions expected to influence species recovery from metabarcoding samples, e.g., GC content (Krehenwinkel et al., 2017), annealing temperature (Krehenwinkel et al., 2018), polymerase (Nichols et al., 2018; Nagai et al., 2022), and other primer sets that match the region of interest for freshwater macroinvertebrate species.” (Lines 225-235).

6. *Line 133: Please add the citation of FastQC: “Andrews, S. (2010). FastQC: A Quality Control Tool for High Throughput Sequence Data”*

**Response:**  The citation for FastQC was added accordingly (Line 109).

7. *Line 142-145: I would slightly disagree with the authors here. Although conceptually very similar, ASVs and OTUS clustered at 100% sequence similarity are not necessarily the same. ASVs/ESVs are the output of a pipeline which includes denoising. ZOTUs could be also just sequences clustered at 100% similarity and this is much less strict than denoising. Since the authors applied a denoising step, I would suggest using the term ASV instead of ZOTUs, since this is the more appropriate term for many people. However, I understand why the term ZOTU was selected and will leave it to the authors to decide that.*

**Response:**  We thank the reviewer for highlighting this point. We agree with the reviewer that different denoising pipelines (i.e., ASV for DADA2 and zOTU for unoise3) could be differentiated, but we still stand that both terms are synonymous in terms of both being “denoising” outputs, with the OUT term used for “clustering” methods. Still, we rephrased the statement to,” Note that sub-OTUs, OTUs with 100% sequence similarity, and zOTUs are synonymous (Edgar, 2016; Porter & Hajibabaei, 2018). Similarly, amplicon sequence variants (ASVs) and exact sequence variants (ESVs) are terms used for outputs of other denoising pipelines (e.g., DADA2 (Callahan et al., 2016)). We used the zOTU in this study since we employed unoise3's algorithm for denoising the quality filtered reads (Edgar, 2016)” (Lines 118-122), adding the points raised by the reviewer regarding the denoising approach. However, we retained the use of “ZOTU” in this study given that we employed unoise3 for the denoising step as previously stated.

8. *Line 155: What exactly is a metabarcoding frequency? I think this should be rather the frequencies of the ZOTUs.*

**Response:**  Rephrased for clarity. Instead of “metabarcoding frequency”, we used, “…normalize the read abundance per sample…” (Lines 129-130).

9. *Results: Line 172: ~500.000 reads assigned to the target ZOTUs over all samples is a relatively low read number, considering the ~15 million reads in total. What happened to the remaining reads? Are these filtered out during bioinformatic processing, in particular denoising? Or are these reads related to contamination? With increasing cycle number, the number of reads in the blanks can be at the order of 10.000. Although this is a considerable amount of contamination, I understand that it is of less concern since the target ZOTU read abundances are very low (if present at all). Please add a statement which explains why you had only ~500.000 target reads in the end.*

**Response:**  A huge number of the raw reads, i.e., 14,239,198 (from the 14.7M) were not assigned to the target zOTUs, because they were filtered out as being erroneous sequences after the denoising step. A supplementary table of the read abundance per step was added in the supplementary files, and the statement discussing the retained 462,665 reads was presented in Lines 145-147, “From the demultiplexed reads, 90% were merged, but only 3% were retained after denoising (Table S1). Most of the reads removed were filtered out being erroneous sequences after the denoising step.”

10. *Discussion: Line 202-204: I don’t understand this sentence because the ZOTUs are shorter than the barcode region.*

**Response:** We truncated and only retained reads that were 421-bp, i.e., the entire BF2/BR2, to not include zOTUs that would be shorter than the barcode length. We rephrased this sentence for clarity, “It is also important to note that we truncated and only retained merged reads with a 421-bp (i.e., the entire length of the BF2 and BR2 barcode region) to prevent the generation of shorter-than-barcode zOTUs for a full-length match against the trimmed Sanger haplotype sequences.” (Lines 180-182).

11. *Line 233-236: I agree to this but want to ask the authors whether the lower detection probability of the ‘rare’ haplotypes could be further reduced by the higher annealing temperature (see comment above) used in this experiment?*

**Response:** We have added a paragraph in the discussion section with regard to this (Response to Comment #5).

12. *Line 267-273: Although this is true, there are many studies out there showing that COI over or underestimates 'true' species diversity (in freshwater macroinvertebrates) when compared to nuclear loci (mito-nuclear discordance). I know that this is a methodological study, but I think it would be beneficial to add a short statement about this issue. Do the authors think that true population genetic analyses can be performed based on a single marker system? For example, K. tibialis showed very high intraspecific diversity based on COI, with a genetic distance of more than 15% for individual haplotypes. Is anything known whether this a cryptic species complex? Or is this rather an artefact of historical isolation, which remains in the mitochondrial genome despite recent gene flow?*

**Response:** We believe that single marker approaches are enough for population genetic analyses as reported by previous literature. We added this statement in the Discussion regarding this comment, “Here, we showed the possibility of generating haplotype information from metabarcoding the mtCOI gene in a mock sample based only on a single gene marker, which could be enough to test or derive population-level hypotheses, e.g., taxa dispersal and distribution at unprecedented scales (Elbrect et al., 2018). Still, given the relatively low cost of metabarcoding, multi-marker assessments can be employed for a more comprehensive population genetic assessment of mixed community samples.” (Lines 257-261).

13. *Figures: Fig. 1: I don’t see the benefit of adding the concentration information in the scale of the circle size AND the number again. This information is much easier to understand from Table 1. I suggest adding the haplotype ID instead of the ‘concentration number’ to the network.*

**Response:**  We thank the reviewer for pointing this out. However, instead of adding the haplotype ID to the network as suggested, we kept this information in the network and revised the concentration scale legend instead. Although we agree with the reviewer that the information is easier to understand from Table 1, we still believe that keeping the concentration number in the network nodes aids in an easier differentiation of each haplotype (Below: Old → New version).

14. *Fig. 2b: This figure has a very low resolution, and the sample abbreviations are hard to read. Why are not all blanks included? Further I would propose to plot first all ‘real samples’ on the left, and then the controls on the right. This would much better show that relative abundances remain stable with increasing cycle number. In the figure description, the word ‘mean’ (line 509) should be lower case.*

**Response:**  Thank you for this. We have updated Fig. 2b and plotted the data as the reviewer suggested, and made sure that the resolution and sample labels are now more readable. Only the blanks with detections were presented in the plot. (Below: Old → New version).

15. *Supplement: The header of Table S2 is not the correct header. Thank you for your paper and all the best!*

**Response:**  Thank you for noticing. Table S2’s header was corrected to “Taxonomic match of the zero-radius operational taxonomic units (ZOTUs) from the DNA metabarcoding sequences against the 658-bp mtCOI Sanger sequences” (Line 316-317).

# Reviewer #2:

(Changes highlighted in **GREEN** font on the revised version of the manuscript)

1. *In their study, "Haplotype-level metabarcoding of freshwater macroinvertebrate species: a prospective tool for population genetics analysis," Serrana and Watanabe explore the potential of using DNA metabarcoding to investigate population genetic patterns at the haplotype level. They conducted experiments on a mock community comprising four species and 20 haplotypes, examining the impact of different DNA concentrations and PCR cycles on the recovery of haplotypes and the detection of true and spurious haplotypes. They employed Sanger-sequenced specimens to create Illumina libraries via a one-step protocol and utilized JAMP for subsequent sequence analysis. Their findings indicate that the limited read length of Illumina sequencing hinders haplotype resolution, as shorter sequences eliminate diagnostic nucleotides. They also discovered that 24-64 PCR cycles recovered 14 assigned ZOTUs, with only 12 ZOTUs having a 100% match to Sanger sequences. The authors conclude by suggesting that: 1) longer read approaches might enable better haplotype detection; 2) the primers used in this study effectively detected targeted species, but alternative primer sets should be tested for potential variations in results; 3) the impact of increased PCR cycles on the relative abundance of quality-passed reads in low-diversity mock samples needs further examination to determine whether this observation extends to more diverse communities; and 4) contamination between libraries and tag-jumping can lead to erroneous read-to-sample assignments, necessitating careful consideration in the development of laboratory protocols and bioinformatics pipelines.*

*While appreciating the effort put into this emerging area of research, the study's purpose is unclear, as DNA metabarcoding for haplotype population genetic analysis is not particularly novel. Additionally, the influence of DNA concentration and PCR cycles on haplotype recovery has been previously explored. The paper's focus on future work and improvements raises concerns about its immediate relevance. In a real community sample, bioinformatic filtering strategies and haplotype mock communities can assist in data cleaning decisions, making the analysis of DNA concentration and PCR cycles less relevant. Furthermore, qPCR for tissue samples could provide more information about naturally occurring DNA concentrations and primer bias. The study's generalizability is also questionable without testing a real environmental sample.*

*To enhance the study's usefulness for a broader audience, it could be reframed to offer a more cost-effective method for studying bulk samples of species compared to Sanger sequencing. This approach could move away from whole community assessments and provide valuable insights for researchers in the field.*

**Response:** We thank the reviewer for the comprehensive summary of our submission. We revised the manuscript accordingly following the insightful feedback of the reviewer on reframing our presentation on the cost-effectiveness of the method for studying bulk samples of specimens compared to Sanger sequencing.

We rephrased the Abstract, “Metabarcoding is a molecular-based tool capable of large quantity high-throughput species identification from bulk samples that is a faster and more cost-effective alternative to conventional DNA-sequencing approaches. Still, further exploration and assessment of the laboratory and bioinformatics strategies are required to unlock the potential of metabarcoding-based inference of haplotype information. In this study, we assessed the inference of freshwater macroinvertebrate haplotypes from metabarcoding data in a mock sample. We also examined the influence of DNA template concentration and PCR cycle on detecting true and spurious haplotypes. We tested this strategy on a mock sample containing twenty individuals from four species with known haplotypes based on the 658-bp Folmer region of the mitochondrial cytochrome c oxidase gene. We recovered fourteen zero-radius operational taxonomic units (zOTUs) of 421-bp length, with twelve zOTUs having a 100% match with the Sanger haplotype sequences. High-quality reads relatively increased with increasing PCR cycles, and the relative abundance of each zOTU was consistent for each cycle. This suggests that increasing the PCR cycles from 24 to 64 did not affect the relative abundance of each zOTU. As metabarcoding becomes more established and laboratory protocols and bioinformatic pipelines are continuously being developed, our study demonstrated the method’s ability to infer intraspecific variability while highlighting the challenges that must be addressed before its eventual application for population genetic studies.” (Lines 2-16).

We added this paragraph in the Introduction section, “DNA-based techniques for species identification, e.g., Sanger sequencing, have been developed for cases where morphology-based identification proved problematic (Hebert et al., 2003). DNA barcodes, i.e., selected short fragments of DNA containing genetic information specific to each organism are used to identify species or higher taxa depending on their level of variability. For example, the typical marker for identifying eukaryotic species is the mitochondrial cytochrome c oxidase I (mtCOI) gene (Hebert et al., 2003). However, conventional DNA barcoding faces practical limitations restricting the method to single specimen analysis, making DNA extraction and sequencing of large-scale samples expensive (Stein et al., 2014). Hence, conventional approaches do not always fulfill the need of ecologists and is not ideal for large quantity high-throughput species identification (Taberlet et al., 2012) … In order to overcome the limitations of processing large numbers of specimens, and with the recent advancements of molecular technologies, researchers tapped next-generation sequencing technologies to allow DNA barcode-based identification to be conducted in a massively parallel manner (Piper et al., 2019).” (Lines 18-28) to highlight the challenges of using DNA barcoding, and the usefulness of metabarcoding for large-scale species identification.

We also added a summary statement in the Conclusion section, “We demonstrate that metabarcoding can be used to infer intraspecific variability and confirm its ability to detect true haplotypes with the classical Sanger method as a basis, showing promise for possible applications in population genetic studies. In particular, we showed that haplotype information could be extracted from mixed community samples of freshwater macroinvertebrates. Quality-passed reads relatively increased with increasing PCR cycle numbers. However, the relative abundance of each zOTU was consistent across the cycle numbers suggesting that increasing the cycles did not affect the relative abundance of quality-passed reads in this low-diversity mock sample. Although conventional population genetics tools, e.g., Sanger sequencing, are used for targeted sequencing of specific genes or regions, metabarcoding is advantageous for studying complex mixtures since it enables high-throughput sequencing allowing massively parallel analysis of many samples. Hence, metabarcoding has a lower per-sample cost than Sanger sequencing (Maggia et al., 2017; Nobile et al., 2019). Still, the overall cost would depend on the number of samples and the sequencing depth required. As metabarcoding becomes more established and laboratory protocols and bioinformatics pipelines are continuously being developed, our study demonstrated that the method could be used to infer intraspecific variability, showing promise for possible applications and highlighting the challenges that need to be addressed before haplotype-level metabarcoding can entirely be used for population genetic applications. This includes further assessment of the laboratory, e.g., amplicon library constriction, PCR conditions, and sequence read processing approaches, e.g., denoising and read filtering steps, needed to confidently recover intraspecific information from metabarcoding data.” (Lines 272-288).

# Reviewer #3:

(Changes highlighted in **PURPLE** font on the revised version of the manuscript)

1. *The manuscript by Serrana and Watanabe presents a study to explore metabarcoding as a tool for extracting haplotype sequences for genetic diversity assessment in freshwater systems. The results show successful application of DNA metabarcoding for this purpose on a small scale mock community study, and also highlights the challenges in bringing this approach to environmental scale. The subject of this manuscript is highly relevant, and the results were generated and analysed thoughtfully and thoroughly. The authors describe the potential and some of the challenges associated with the DNA-based haplotype analysis approach, but little discussion is paid to the path forward.*

*My greatest concern with the study is the size and diversity of the utilised mock community, and its ability to highlight the possibilities of using metabarcoding for this purpose, but I recognise that the results look promising, and can be used carefully.*

**Response:**  We are thankful for the reviewer’s good remarks, comments, and feedback on our manuscript. This version has been revised according to the helpful comments of the reviewer.

2. *Specific comments: The mock community is small (4 species, total specimens = 20), and has low taxonomic diversity (3 stonefly and 1 mayfly species). Why did the authors decide on these species? Increased taxonomic diversity in the mock community as well as number of species would have added to the robustness of the results generated.*

**Response:**  We agree with the reviewer’s comment on the low diversity of our samples. We chose these 4 species to form the mock sample for this study mainly due to three reasons. (1) Each individual sample was morphologically identified by our expert taxonomist for this group of insects, with the *Amphinemura decemseta* samples published (Gamboa et al., 2019), and (2) they have enough and good quality DNA. Lastly, (3) the haplotypes we have selected for each species had variable nucleotide diversity which would be good for the assessment. Adde to the text, “Each individual sample was morphologically identified accordingly, and the selected haplotypes for each species had variable nucleotide diversity that would be good for this assessment.” (Lines 73-74).

3. *L129: Which sequencing kit was used?*

**Response:**  The “MiSeq Reagent v3 600-cycle Kit (2 × 300 cycles)” was used and added in the text (Line 107-108).

4. L162: Truncating reads to 421 bp merged some of the haplotypes. Was this an expected result? Did the authors perform an in-silico primer analysis based on the known haplotypes? Could the use of a different DNA barcode have prevented this?

**Response:**  The merging of some Sanger haplotypes after truncating the reads to 421 bp was expected given the loss of some polymorphic sites after shortening the 658 COI barcode as stated in the results (Lines 137-140), “After trimming the Sanger sequences into the 421-bp length of the BF2 and BR2 barcode region to complement the amplified region in the DNA metabarcoding data, three of the K. tibialis Sanger haplotypes (i.e., KT1, KT3, and KT4) were grouped into one haplotype, similar to the two haplotypes of A. decemseta (i.e., AD1 and AD3).”.

We did not perform *in silico* primer analysis based on the known haplotypes since we ought to use the BR2 & BF2 primer sets optimized for freshwater macroinvertebrate species and for metabarcoding analysis. The use of a different barcode couldn’t prevent this given that the nucleotide positions of each Sanger haplotype of *K. tibialis* and *A. decemseta* were dispersed throughout the COI barcode. Still, this was a good point to present the limitations in using metabarcoding data compared to Sanger sequencing as we have discussed in Lines 184-189, “Some species lost nucleotide information, i.e., polymorphic sites, to distinguish the haplotypes due to shortening the barcode region (658-bp to 421-bp). For example, the *K. tibialis* haplotype with a relatively high input DNA template concentration (i.e., 0.10 ng/μL) was likely not a false negative detection. Its reads were present in the DNA metabarcoding data but merged with the other *K. tibialis* haplotype. This highlights the inability of shorter marker regions to differentiate haplotypes of certain species, which requires further development and evaluation.”.

5. *L188: Some of the expected haplotypes were not detected at all. The authors do not discuss this result further. It would be nice to have some discussion about how to proceed with overcoming this challenge.*

**Response:** Additional discussion for the non-detections, and the possible influence of annealing temperature was added in the Discussion section (Lines 225-235). See response to Reviewer #1 - Comment #5 for more information.

6. *L218: The presented statistics about Nanopore data are out of date, expectations of data quality have improved since chemistry R9.*

**Response:** We thank the reviewer for this remark. We have updated the statement in this version, “Still, given the raw read error rate from these long-read sequencing platforms, i.e., around 6% for quality scores at least equal to 10 for the Oxford Nanopore MinIONTM (Delahaye & Nicolas, 2021)…” (Lines 195-196).

7. *L228: The authors state that eliminating false or artificial haplotypes will be difficult, but is there any idea about how this challenge could be addressed going forward?*

**Response:** Additional discussion for eliminating false and artificial haplotypes going forward was added in the Discussion section, “Identifying and eliminating artificial or false haplotypes has been a major challenge for population-level inference from metabarcoding data... Macé et al. (2022) suggested that a denoising method with an additional bimeric sequence removal step combined with a specific polymorphic mitochondrial barcode might resolve the issue of false haplotype detection. In addition, read filtering by relative abundance could help remove false positives and chimeras given that these sequences are usually low in abundance (Macé et al., 2022). Some studies have performed additional read filtering steps based on haplotype presence rate for different PCR replicates (Tsuji et al., 2020a,b). However, these previous resolutions might lead to removing true haplotypes in the community sample with lower abundance. Hence, additional assessment and development of read processing are warranted moving forward.” (Lines 204-214).

8. *The discussion could be improved further by the inclusion of a closing paragraph that sums up the perspectives and recommendations to develop the approach further toward implementation on environmental samples used freshwater assessments.*

**Response:** We thank the reviewer for this remark, a paragraph regarding this was added in the Discussion section for *DNA metabarcoding for haplotype-level inference*. Please refer to Reviewer #2’s response on this.

# References

Andrews, S. (2010). FastQC: a quality control tool for high throughput sequence data.

Callahan, B. J., McMurdie, P. J., Rosen, M. J., Han, A. W., Johnson, A. J. A., & Holmes, S. P. (2016). DADA2: High-resolution sample inference from Illumina amplicon data. *Nature Methods*, 13(7), 581-583.

Delahaye, C., & Nicolas, J. (2021). Sequencing DNA with nanopores: Troubles and biases. *PloS One*, 16(10), e0257521.

Edgar, R. C. (2016). UNOISE2: improved error-correction for Illumina 16S and ITS amplicon sequencing. *bioRxiv*, 081257.

Elbrecht, V., Braukmann, T. W., Ivanova, N. V., Prosser, S. W., Hajibabaei, M., Wright, M., ... & Steinke, D. (2019). Validation of COI metabarcoding primers for terrestrial arthropods. *PeerJ*, 7, e7745.

Gamboa, M., Muranyi, D., Kanmori, S., & Watanabe, K. (2019). Molecular phylogeny and diversification timing of the Nemouridae family (Insecta, Plecoptera) in the Japanese Archipelago. *PloS One*, 14(1), e0210269.

Hebert, P. D., Cywinska, A., Ball, S. L., & Dewaard, J. R. (2003). Biological identifications through DNA barcodes. *Proceedings of the Royal Society of London. Series B: Biological Sciences*, 270(1512), 313-321.

Krehenwinkel, H., Wolf, M., Lim, J. Y., Rominger, A. J., Simison, W. B., & Gillespie, R. G. (2017). Estimating and mitigating amplification bias in qualitative and quantitative arthropod metabarcoding. *Scientific Reports*, 7(1), 1-12.

Krehenwinkel, H., Fong, M., Kennedy, S., Huang, E. G., Noriyuki, S., Cayetano, L., & Gillespie, R. (2018). The effect of DNA degradation bias in passive sampling devices on metabarcoding studies of arthropod communities and their associated microbiota. *PLoS one*, 13(1), e0189188.

Leese, F., Sander, M., Buchner, D., Elbrecht, V., Haase, P., & Zizka, V. M. (2021). Improved freshwater macroinvertebrate detection from environmental DNA through minimized nontarget amplification. *Environmental DNA*, 3(1), 261-276.

Macé, B., Hocdé, R., Marques, V., Guerin, P. E., Valentini, A., Arnal, V., ... & Manel, S. (2022). Evaluating bioinformatics pipelines for population‐level inference using environmental DNA. *Environmental DNA*, 4(3), 674-686.

Maggia, M. E., Vigouroux, Y., Renno, J. F., Duponchelle, F., Desmarais, E., Núñez, J., ... & Mariac, C. (2017). DNA metabarcoding of Amazonian ichthyoplankton swarms. *PLoS One*, 12(1), e0170009.

Nagai, S., Sildever, S., Nishi, N., Tazawa, S., Basti, L., Kobayashi, T., & Ishino, Y. (2022). Comparing PCR-generated artifacts of different polymerases for improved accuracy of DNA metabarcoding. *Metabarcoding and Metagenomics*, 6, e77704.

Nichols, R. V., Vollmers, C., Newsom, L. A., Wang, Y., Heintzman, P. D., Leighton, M., ... & Shapiro, B. (2018). Minimizing polymerase biases in metabarcoding. *Molecular Ecology Resources*, 18(5), 927-939.

Nobile, A. B., Freitas-Souza, D., Ruiz-Ruano, F. J., Nobile, M. L. M., Costa, G. O., De Lima, F. P., ... & Oliveira, C. (2019). DNA metabarcoding of Neotropical ichthyoplankton: Enabling high accuracy with lower cost. *Metabarcoding and Metagenomics*, 3, e35060.

Porter, T. M., & Hajibabaei, M. (2018). Scaling up: A guide to high‐throughput genomic approaches for biodiversity analysis. *Molecular Ecology*, 27(2), 313-338.

Stein, E. D., Martinez, M. C., Stiles, S., Miller, P. E., & Zakharov, E. V. (2014). Is DNA barcoding actually cheaper and faster than traditional morphological methods: results from a survey of freshwater bioassessment efforts in the United States? *PloS One*, 9(4), e95525.

Tsuji, S., Maruyama, A., Miya, M., Ushio, M., Sato, H., Minamoto, T., & Yamanaka, H. (2020a). Environmental DNA analysis shows high potential as a tool for estimating intraspecific genetic diversity in a wild fish population. *Molecular Ecology Resources*, 20(5), 1248-1258.

Tsuji, S., Shibata, N., Sawada, H., & Ushio, M. (2020b). Quantitative evaluation of intraspecific genetic diversity in a natural fish population using environmental DNA analysis. *Molecular Ecology Resources*, 20(5), 1323-1332.

Zizka, V. M., Elbrecht, V., Macher, J. N., & Leese, F. (2019). Assessing the influence of sample tagging and library preparation on DNA metabarcoding. *Molecular Ecology Resources*, 19(4), 893-899.
